# Physician–patient communication in rheumatology: a systematic review

**DOI:** 10.1007/s00296-018-4016-2

**Published:** 2018-03-26

**Authors:** Sofia Georgopoulou, Louise Prothero, David P. D’Cruz

**Affiliations:** 1 Department of Psychology, University of East London, Arthur Edwards Building, Water Lane, Stratford, E15 4LZ UK; 2 Clinical Trials Group, Academic Department of Rheumatology, King’s College London, Faculty of Life Sciences and Medicine, 3rd Floor Weston Education Centre, 10 Cutcombe Road, Denmark Hill, London, SE5 9RJ UK; 3 Louise Coote Lupus Unit, 4th Floor Tower Wing, Guys Hospital, London, SE1 9RT UK

**Keywords:** Physician–patient communication, Doctor–patient interaction, Rheumatic diseases, Patient outcomes

## Abstract

The nature of physician–patient interaction can have a significant impact on patient outcomes through information-sharing and disease-specific education that can enhance patients’ active involvement in their care. The aim of this systematic review was to examine all the empirical evidence pertaining to aspects of physician–patient communication and its impact on patient outcomes. A systematic search of five electronic databases (MEDLINE, PsycINFO, EMBASE, CINAHL, and Web of Science) was undertaken from earliest record to December 2016. Studies were eligible if they: (1) included adult participants (18 years or over) with a diagnosis of a rheumatic condition; (2) were of quantitative, qualitative or mixed methods design; (4) were surveys, observational and interventional studies; (5) were published in the English language; and (6) reported findings on either various physician–patient communication aspects alone or in combination with physical and psychological outcomes. Searches identified 455 papers. Following full-text retrieval and assessment for eligibility and quality, ten studies were included in the review; six quantitative, one mixed methods, and three qualitative papers. Higher levels of trust in the physician and active patient participation in the medical consultation were linked to lower disease activity, better global health, less organ damage accrual, greater treatment satisfaction with fewer side effects from the medication, more positive beliefs about control over the disease, and about current and future health. Future research could focus on the design and implementation of interventions incorporating communications skills and patient-education training.

## Introduction

Physician–patient communication is a central clinical function that is complex in nature and has received increased attention in recent years. The main goals of the interaction between doctor and patient are: information sharing to support diagnosis and treatment, relationship building and disease-specific education. In this process, doctors’ communication style, attitudes, beliefs, and perceptions can influence the relationship dynamic and enhance patients’ active involvement in their care which may lead to improved outcomes [1, 2].

Studies involving patients with rheumatic diseases suggest that the quality of communication with doctors is linked to patient outcomes. For instance, Fawole et al. reported that the nature of physician–patient interactions in the assessment and management of patients with rheumatic diseases can have a significant impact on patients’ health-related quality of life (HRQoL) [2]. One of the domains that can be affected by the quality of contact with the health care provider and the amount of information provided to patients by the quality of this interaction is medication adherence [3]. Medication adherence ranges between 30–99% in patients with rheumatic diseases and is multifactorial [4]. Physician behaviour can have a considerable impact on relationship building and development of trust with patients which can affect adherence [5]. If there is lack of concordance and trust in the physician and insufficient information, the likelihood of the patient being non-adherent to the medication is high [6] increasing the risk for greater disease activity, flares and organ damage. Education level and medication side effects can also affect medication adherence [7].

Additional factors that can have a significant impact on patient outcomes particularly on their risk of cardiovascular disease (CVD) include traditional and lifestyle risk factors such as smoking, hypertension, hypercholesterolemia, diabetes, and physical inactivity [8, 9]. Existing literature is indicative of the fact that inflammatory rheumatic diseases are linked to an increased risk of cardiovascular disease (CVD) [10] with fatal and nonfatal CVD being higher in patients with RA and SLE compared to the general population. A healthy lifestyle such as following a balanced diet, stopping smoking, losing weight, and increasing physical activity levels can, therefore, decrease the risk of CVD in these conditions. Communicating this effectively to patients and supporting them in modifying aspects of their lifestyle requires that the relationship between doctor and patient be characterised by trust and good rapport [11]. Communication is vital to patients’ understanding of their illness and the risks and benefits associated with its treatment [12] and a vital component in developing and maintaining a relationship that involves support, empathy, understanding [13], good collaboration [14], and patient-centred interviewing all of which can enhance outcomes such as adherence, diet, smoking cessation, and physical activity levels.

Although there have been reviews on physician–patient communication, they generally refer to the topic across different conditions and do not focus exclusively on rheumatic diseases [15, 16]. In addition, they are narrative reviews rather than systematic reviews and they were conducted over a decade ago [16, 17]. This highlighted the need for a review to systematically identify and appraise the literature on doctor–patient communication with a specific focus on patients with rheumatic diseases. Data on the quality of interaction between patients and clinicians in rheumatology could identify potential factors that can be addressed to improve communication—if the evidence suggests a need for it. As a second step, based on the review findings, the ground could be set for the design and implementation of a project that could explore the efficacy of an intervention involving training rheumatology health care professionals in motivational interviewing (MI) with the aim to enhance communication with their patients and improve outcomes.

The current review aimed to systematically identify, appraise and evaluate the evidence on: (a) factors influencing physician–patient communication and (b) the association between doctor–patient interaction and health outcomes.

## Methods

### Search strategy

Literature searches and the design of the current article were both performed according to previously published recommended considerations for writing a narrative biomedical review to maximise the robustness and impact of this review [18]. Thus, a systematic electronic search of peer-reviewed studies published in the English language from inception to December 2016 was undertaken in five standard bibliographic databases (MEDLINE, PsycINFO, EMBASE, CINAHL, and Web of Science) using combinations and MESH terms of the following keywords : rheumatic disease, systemic lupus erythematosus, rheumatology, patient physician interaction, work alliance, patient–physician communication, physician–patient relationship, provider–patient relationship, doctor patient relationship, or doctor–patient communication, provider–patient communication, patient outcomes, health-related quality of life, and well-being (Table 1). Reference lists of relevant included papers were further screened for additional relevant studies that might have been missed by the electronic database searches.

**Table 1:** Search strategy overview

Databases searched
MEDLINE, EMBASE, PsycINFO, CINAHL, Web of Science
Rheumatic disease/ or systemic lupus erythematosus/ or SLE/ or rheumatology/ or rheum* (incl. MESH terms/exploding terms)
Patient physician interaction/ or work alliance/ or patient–physician communication/ or physician–patient relationship/ or provider–patient relationship/ or doctor patient relationship/ or doctor–patient communication/ or provider–patient communication
Patient outcomes/ or health-related quality of life/ or well-being
1 and 2
1 and 2 and/or 3

### Eligibility criteria

Observational or interventional studies of qualitative, quantitative or mixed-method design or RCTs examining physician–patient communication and associated outcomes for adult patients (≥ 18 years) with all kinds of rheumatic diseases, published in English in peer-reviewed journals were considered for inclusion (Table 2). No limitations were made regarding clinical outcomes.

**Table 2:** Inclusion criteria

Inclusion criteria (general)
Types of studies
Qualitative or quantitative studies
Observational studies or RCTs
Types of participants
Adult patients with one or more rheumatic disease(s) of either non-inflammatory nature (e.g., fibromyalgia syndrome) or of inflammatory nature (e.g., RA, SLE, AS) or both
Inclusion criteria (specific)
Communication
Studies exploring (and reporting statistical results—if applicable) aspects of communication or interaction between physician and patient such as trust, decision-making, explanation, education, knowledge, respect, understanding, patient involvement and participation etc
AND/OR
Outcomes
Studies assessing outcomes such as (but not limited to) disease activity, morbidity, psychological/physical status, medication adherence, organ damage, treatment satisfaction associated with physician–patient communication components

### Exclusion criteria

Unpublished data in the form of conference abstracts and university theses were excluded from the review.

### Selection of studies

Identified papers were screened for eligibility by the primary author (SG) based on title and abstracts followed by reference screening of studies that were deemed eligible. After full-text retrieval, additional, independent review of the included papers by the authors (LP and DPD’C) was performed to confirm compliance with study inclusion criteria. Consensus was used to resolve differences.

### Data extraction and management

Study data were extracted by the primary review author (SG) using a predesigned data extraction form on the following details: (1) aim; (2) country; (3) design and methods; (4) sample size and diagnosis; (5) description of communication/patient outcome variables; and (6) conclusions (see Tables 3, 4).

**Table 3:** Overview of quantitative studies included in the systematic review

Study	Aim	Country	Design	Sample size	Communication/outcome variable(s)	Conclusion
Street et al. [23]	To examine the extent of influencing factors on medical interactions in patients with SLE e.g., patient characteristics, physician communication style	USA	Cross-sectional examination of audio-recorded consultations	79 SLE	Verbal behaviour coded as active patient participation (e.g., questions, assertive responses, negative emotions, expressions of concern) and physician partnership building (e.g., encouragement, affirmations, reassurance)	Patients were more active participants when interacting with physicians who more frequently engaged in partnership building and supportive talk (adj. *m* = 11.21; 95% CI 5.73–16.69; *p* < 0.001). Higher education level predicted and being white predicted more active participation
Beusterien et al. [24]	To assess relationships between physician–patient relationship and patient outcomes including health status and regimen satisfaction in SLE	USA	Cross-sectional survey	302 SLE	Patients’ perceptions of treatment regimen and satisfaction, physician–patient interactions including involvement in treatment decisions, physician bedside manner, satisfaction with physician, SLE control and severity, current health and hope about future health	Positive physician–patient interactions led to higher satisfaction with treatment regimen and feeling well-controlled and more favourable perceptions of current health and being more hopeful about future health (*t* = 6.10; *t* = 4.07)
Ward et al. [25]	To examine associations between active patient–physician communication and measures of morbidity in patients with SLE	USA	Cross-sectional questionnaire study	79 SLE	Patient communication behaviours coded as question asking, assertive responses, expressions of concern. Outcome measures assessed: depression (CES-D), SLE activity and morbidity SLAM, SLEDAI, SLICC/ACR Damage Index, Health Assessment Questionnaire (HAQ)	Patients who participated more actively in their visits with physicians had less cumulative organ damage due to SLE. With each additional 1-point increase in active patient participation score, Damage Index decreased on average by 7% (OR = 0.93; 95%CI 0.91–0.94; *p* < 0.0001)
Freburger et al. [26]	To assess the psychometric properties of the Trust in Physician Scale and to identify variables associated with patient trust in their rheumatologist	USA	Cross-sectional questionnaire survey	713 (39% OA; 47% RA; 37% FM)	Trust in Physician Scale, questions on self-rated health and comorbidities, Modified Health Assessment Questionnaire (MHAQ), questions on medical skepticism and decision-making	Fairly high level of trust in rheumatologists (mean = 76.25; SD = 13.29). Patients with poorer health reported lower levels of trust than those with better health (*r* = − 0.10; *p* < 0.05). FM and OA patients had less trust in their rheumatologist than those with RA (*β* = − 4.58; *p* < 0.001 and *β* = − 3.66; *p* = 0.003)
Berrios-Rivera et al. [27]	To identify components of the patient–doctor relationship associated with trust in physicians	USA	Cross-sectional questionnaire survey	102 (70 RA; 32 SLE)	Hall’s Trust in Physicians Scale, Kaplan’s Physicians’ Participatory Decision-making Style Scale on doctors’ informativeness, doctors’ sensitivity to concerns, doctors’ reassurance and support and patient-centred behaviour	Satisfaction with medical care interaction and trust was moderate (6.2–7.1) as to patient–doctor communication. Increased trust was correlated with fewer side effects (*r* = − 0.28; *p* < 0.05) and better global health (*r* = 0.20; *p* < 0.05). Patient-centred communication associated with patient disclosure of information (*B* = 0.38; *p* < 0.001) which, in turn, was negatively associated with disease activity (*B* = − 0.20; *p* = 0.03)
Ishikawa et al. [28]	To examine how patient preferences for decision-making affect the relationship between their participation style in visit communication and the feeling of being understood	Japan	Cross-sectional questionnaire study	115 RA	Autonomy Preference Index for autonomous decision-making, self-reported patient participation, patient feelings of being understood, Arthritis Impact Measurement Scales 2	Patients were more likely to feel understood by the physician when they perceived that they had more actively participated in visit communication (*r* = 0.36; *p* < 0.001). Increased shared decision-making was linked to better physician–patient communication

**Table 4:** Overview of qualitative studies included in the systematic review

Study	Aim	Country	Design	Sample Size	Communication/outcome variable(s)	Conclusion
Ahlmén et al. [29]	To explore the most important outcomes for patients’ RA treatments, decisions relating to when a treatment is working, and factors associated with treatment satisfaction/dissatisfaction	Sweden	Qualitative/focus groups	25 RA	Physical and psychosocial items which comprised overall treatment goals such as impairment in social roles, fatigue, daily activities and self-confidence	Identified themes were: “normal life”, “physical capacity”, “independence”, and “well-being”. Satisfaction with treatment was linked to quality of communication between rheumatology staff and patients, which was regarded as a pre-requisite for effective treatment. Patients wanted to be accepted as experts on their own bodies, and expected all clinicians to be experts in RA which made it possible for them to “take charge” of their lives
Haugli et al. [30]	To evaluate what patients with rheumatic disease perceive as important in their medical encounters	Norway	Qualitative/focus groups	26 (12 RA/AS; 14 FM)	Questions on patient experiences with providers related to their present illness, questions on what patients experienced as the most important aspect of their provider–patient relationship, and more specific questions on if and how physicians are helpful in coping with their illness, or if the relationship could be stressful	Two central themes emerged as important: “to be seen” and “to be believed”. For RA and AS patients, this meant to be seen as an individual and not as a mere diagnosis, and to be believed as far as pain and suffering was concerned. For FM patients, both themes referred to being able to obtain a useful somatic diagnosis. Reciprocal trust, availability of the physician and receiving adequate information about the disease and the diagnosis were further concepts mentioned
Chambers et al. [31]	To explore why patients with SLE did or did not take their medications as prescribed	UK	Qualitative interviews	33 SLE	Questions on what medications for SLE were prescribed and if patients always take them, patient understanding of the reasons they take the medications and any difficulty obtaining them, questions on SLE causes, cultural and religious beliefs, use of complementary and alternative therapies	Reasons for taking medications: fear of worsening disease, belief of no effective therapeutic alternative, lack of knowledge about SLE to allow confidence in changing medications and feelings of moral obligation or responsibility to other. Themes for not taking medications: belief in alternative methods, belief that long-term use of drugs was unnecessary, fear of drug adverse effects, practical difficulties in obtaining medications, and poor communication with doctors
Koneru et al. [32]	To measure levels of adherence to medications in SLE patients, to assess the relevance of risk factors of non-adherence for SLE and other rheumatic diseases	USA	Mixed methods	63 SLE	Patient understanding of their disease and its severity, attitudes towards effectiveness and side effects of medications, trust in the SLE health care providers, understanding of instructions and rationale for medical interventions, comorbidities, use of CAMs. Outcome measures: Beck’s Depression Inventory (BDI), Religiosity Commitment Inventory-10, MOS-SF36, Systemic Lupus Activity Questionnaire (SLAQ), SLICC/ACR Damage Index, measure of medication adherence	Modest adherence to medications. 61% to prednisone, 49% to hydroxychloroquine, 57% to immunosuppressants. Risk factors for non-adherence included single status, complicated medication regimens, limited comprehension of physician explanations and instructions (*χ*^2^ = 10.70; *p* = 0.001; OR = 26.20; 95% CI 7.30–95; *R*^2^ = 48%) and having to take medications more than once daily. Busy lifestyle, and forgetting to take their medications were barriers to adherence whereas pill boxes were most helpful for adhering to medication

### Assessment of quality of reporting and methodological quality

Two different instruments were used to assess methodological quality of the studies due to the inclusion of both quantitative and qualitative papers in the current review to provide a robust evaluation.

The Newcastle–Ottawa Quality Assessment Scale (NOS) [19] adapted for cross-sectional studies [20] was selected to assess quality of the quantitative studies. A total score based on the degree of appropriateness of the research design, recruitment strategy and response rate, representativeness of participants, objectivity/reliability of outcome measures, power calculations, and appropriateness of statistical analyses was computed to assess methodological quality.

The adapted for cross-sectional studies NOS contains seven items, categorized into three dimensions (selection, comparability, and outcome). For each item, a series of response options is provided allowing to score a maximum total of ten stars for assessment of study quality across all questions.

The Critical Appraisals Skills Programme (CASP) criteria [21] was chosen as a quality assessment instrument for the qualitative studies due to its inclusivity in addressing different aspects of study quality, including methodological and theoretical aspects [22]. Its broad checklist-type quality criteria offer a flexible way to assess rigour, credibility and relevance despite the heterogeneous nature of included studies. Studies are evaluated based on congruency between the paradigm, methodology and method, the influence of the researcher on the research, data collection and data analysis. A maximum of 100% can be scored across ten questions each of which includes a series of response options which need to have been addressed in the study.

Independent assessment of the risk of bias of each of the included studies was undertaken by two reviewers (SG & LP). Each study was classified as “excellent”, “good”, “acceptable”, or “weak” based on the total methodological quality assessment score which was the sum of individual domains scores (see Tables 5, 6). Score disagreements were discussed and resolved by consensus through allocation of a final agreed rating for each study which was confirmed by the third reviewer (DPD’C).

**Table 5:** Overview of quality assessment scores—cross-sectional studies (Newcastle–Ottawa Scale—NOS)

Authors	Mean NOS Score (reviewers 1 and 2)	Quality Assessment Category
Street et al. [23]	8.5* (8* and 9*)	Excellent
Beusterien et al. [24]	5.5* (5* and 6*)	Acceptable
Ward et al. [25]	10* (10* and 10*)	Excellent
Freburger et al. [26]	9* (9* and 9*)	Excellent
Ishikawa et al. [28]	8.5* (8* and 9*)	Excellent
Berrios-Rivera et al. [27]	8* (8* and 8*)	Good
Koneru et al. [32]	9* (9* and 9*)	Excellent

*Quality Assessment Categories: < 5 (weak); 5–6.5 (acceptable); 6.6–8 (good); 8.1–10 excellent

**Table 6:** Overview of quality assessment scores—qualitative studies (Critical Appraisals Skills Programme—CASP)

Authors	Mean CASP Score (reviewers 1 and 2)	Quality Assessment Category
Ahlmén et al. [29]	91% (90 and 92%)	Excellent
Haugli et al. [30]	96% (98 and 94%)	Excellent
Chambers et al. [31]	98% (98 and 98%)	Excellent

*Quality Assessment Categories: < 50 (weak); 50–65 (acceptable); 66–80 (good); 81–100 (excellent)

## Results

### Selection process

**Fig. 1:**
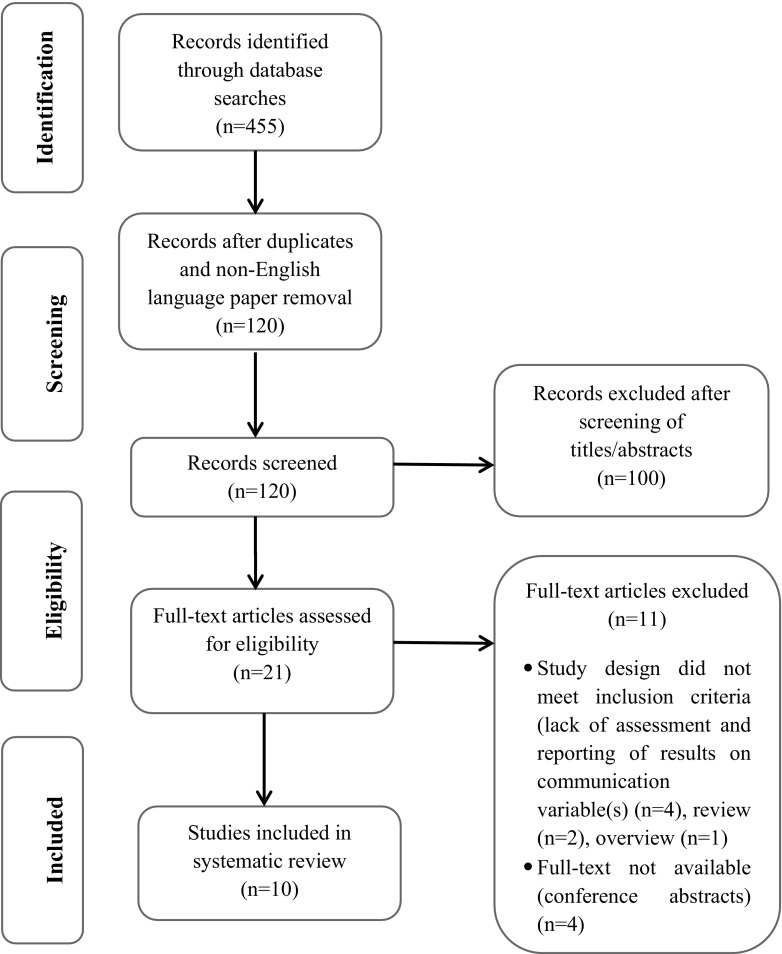
Flowchart of selection process

The searches identified 455 publications. 21 of these were examined in full text, of which ten studies [23–32] met the inclusion criteria (Fig. 1). Excluded studies were due to lack of fulfilment of eligibility criteria. Four of the excluded articles [33–35] were conference abstracts and published full-text papers were not available, two were literature reviews [16, 36], four did not assess communication or outcome variables [37–40], and one was a description of educational programmes efficacy regarding patients with rheumatoid arthritis [17]. Quality criteria were met with ‘excellent’ mean quality assessment scores for both quantitative and qualitative studies (8.4* and 95%, respectively) (see Tables 5, 6).

### Overall description of included studies

The characteristics of the included studies are shown in Tables 3 and 4. The studies were published between 2003 and 2013. Six were of quantitative design, three were qualitative and one employed mixed methods. The majority of the quantitative studies [23–27] were conducted in the USA, whereas one was done in Japan [28]. The qualitative studies took place in Sweden [29], Norway [30] and the UK [31], while the mixed-method study was conducted in the USA [32].

### Description of quantitative studies

Two main themes emerged from the quantitative papers that were identified in this review: (a) the factors associated with better communication between physicians and patients and (b) the impact of the quality of this interaction on patient outcomes.

Overall, based on the evidence from the identified papers, it appeared that: (a) greater patient participation, shared decision-making, treatment satisfaction and trust in the physician were associated with better communication between physician and patient [23, 25, 28]. Higher quality physician–patient communication, in turn, was associated with better global health, less organ damage, lower disease activity, and fewer medication side effects [23–28].

Patients who participated more actively in their interaction with physicians tended to have less permanent organ damage [25]. The authors reported a dose–response relationship with the average of the SLICC/ACR Damage Index decreasing by 7% for every additional one-point increase in the patient active communication score (OR = 0.93; 95% CI 0.91–0.94; *p* < 0.0001). Although the analysis was cross-sectional, the study had longitudinal components suggesting a possibility that active communication may affect the development of future complications in patients with SLE. Ward et al. emphasized that socioeconomic status (SES) could not explain this association, since it was correlated neither with participatory communication (education level: *r* = − 0.08; *p* = 0.49; Hollingshead Index: *r* = 0.00; *p* = 0.99) nor with physician patient-centred communication (education level: *r* = − 0.10; *p* = 0.37; Hollingshead Index: *r* = 0.05; *p* = 0.69).

Street et al. [43] reported that what seemed to have been associated with increased patient participation in interactions with physicians was more frequent engagement in partnership building (adj. mean = 3.36; 95% CI 2.02–4.69; *p* < 0.01) and supportive talk on behalf of the physician (adj. mean = 1.41; 95% CI 0.49–2.33; *p* < 0.01) in patients with SLE. The authors highlighted the fact that a variety of contextual as well as patient and clinician factors could be linked to the quality of the interaction. Clinician communication style and the clinical setting such as time allotted for the consultation were predictive of patient participation in medical consultations [23].

The impact of increased patient participation in the medical consultation was described by Ishikawa et al. [28]. Patients with RA who were more active participants reported feelings of being understood by the physician (*r* = 0.36; *p* < 0.001) and increased shared decision-making was linked to better physician–patient communication [28]. However, feelings of being understood depended on patients’ decision-making preferences. For example, patients who were more autonomous in decision-making reported feelings of being understood if they were more active in the consultation, while patients who had lower preference for decision-making did not (preference × participation: *β* = 0.089, SE = 0.030; *p* = 0.021). The authors suggested that these findings may be influenced by Japanese culture, since Japanese patients are more focused on seeking information to build rapport with the physician rather than making decisions [41].

Beusterien et al. [24] reported that positive physician–patient interactions led to higher satisfaction with treatment regimen and feeling well-controlled (*t* = 6.10), less depressed (*t* = 7.19) and more favourable perceptions of current health (*t* = 4.07) [24]. Goal-setting, in particular was associated with being more hopeful about future health in patients with SLE (*t* = 3.02).

Freburger et al. [26] found that lower levels of trust in physicians were linked to poorer health (*r* = − 0.10; *p* < 0.05). Patients reported having fairly high levels of trust in rheumatologists (mean = 76.25; SD = 13.29). This, however, was dependent on the condition that patients had. For example, patients with FM and osteoarthritis (OA) had less trust in their rheumatologist than patients with RA (*β* = − 4.58; *p* < 0.001 and *β* = − 3.66; *p* = 0.003) [26]. Other variables such as older age (*r* = − 0.129; *p* = 0.002), minority status (*r* = 2.708; *p* = 0.05), and higher education level (*r* = − 0.332; *p* = 0.047) were also associated with lower levels of trust in physicians.

According to Berrios-Rivera et al. [27], increased trust but also additional components of the medical interaction such as informativeness, patient-centredness, sensitivity to patient concerns, and disclosure of information were correlated with fewer side effects (*r* = − 0.30; *p* < 0.005; *r* = − 0.27; *p* < 0.05; *r* = − 0.24; *p* < 0.05; *r* = − 0.29; *p* < 0.05) and better global health (*r* = 0.20; *p* < 0.05), respectively. Results in patients with RA and SLE also showed that the association between trust and medical care interaction was moderate (6.2–7.1) regarding patient–doctor communication, which was suggestive of the involvement of the other components. Communication that focused on the patient was related to patient disclosure of information (*B* = 0.38; *p* < 0.001) which, in turn, was negatively associated with disease activity (*B* = − 0.20; *p* = 0.03) [27]. The authors emphasized that in patients with RA and SLE, ethnicity was an important factor associated with trust in physicians (African-American: *B* = − 0.64; *p* = 0.005; Latino: *B* = − 0.29; *p* = 0.001).

### Description of qualitative studies

The three qualitative papers [29–31] and the one mixed-method study [32] included in this review could be classified into two thematic categories: (a) important outcomes of treatment and of the medical consultation [29, 30] and (b) factors associated with medication intake relative to the nature of physician–patient communication [31, 32].

Ahlmén et al. [29] conducted focus groups with patients who had RA. A number of themes emerged from the interviews such as: “normal life”, “physical capacity”, “independence”, and “well-being”. Patient aims included regaining full health and having a normal life without limitations and not be considered as different by people because of their condition and associated disability. “Physical capacity” referred to patients’ loss of functionality and improvement in symptoms such as fatigue, pain and stiffness, muscle strength were reasons to make them feel happy. Patients wanted to be able to be independent and manage daily activities such as personal hygiene, walking and dressing as well as work and social activities. “Well-being” was a concept that patients could not clearly define but was rated as an important outcome in terms of feeling happy, enjoying life and regaining self-confidence. Satisfaction with treatment was linked to quality of communication between rheumatology staff and patients, which was regarded as a pre-requisite for effective treatment. A good relationship with the clinician was also related to mutual respect and trust. Patients expected all clinicians to be experts in RA but wanted to be accepted as experts on their own bodies, which made it possible for them to “take charge” of their lives [29].

Haugli et al. [30] interviewed two groups of patients: one with well-defined inflammatory conditions such as RA or ankylosing spondylitis (AS) and one with non-inflammatory conditions such as FM. The relationship with their doctor was rated as important by patients in both groups. Two central themes emerged from the interviews: patients wanted “to be seen” and “to be believed”. For RA and AS patients, this meant to be seen as an individual and not as a mere diagnosis, and to be believed as far as pain and suffering was concerned. “To be seen” also had implications for the physician’s ability to take into consideration the perspective of the patient and to provide them with sufficient information about the condition and the prognosis. “Being believed” also suggested that the doctor acknowledged patients’ expert knowledge and the jointly agreed development of a treatment plan. For FM patients, both themes referred to being able to obtain a useful somatic diagnosis. According to patients, receiving a diagnosis meant being believed, because “no objective findings” implied no “real” disease which was associated with patients feeling that they were mistrusted by the physician. Getting the right diagnosis was also interlinked with the necessary investigations, appropriate treatment, and information provision regarding the disease. Reciprocal trust, availability of the physician, and receiving adequate information about the disease and the diagnosis were further concepts mentioned. All patient groups reported that the relationship with their doctor was important in the care of their health problems [30].

Chambers et al. [31] interviewed patients with SLE to explore reasons for medication adherence. The reasons cited by patients for taking their medications were: (a) fear of worsening disease especially in patients who had experienced life-threatening episodes of SLE; (b) the belief that there was no other effective therapeutic alternative to Western medicines; (c) lack of knowledge about SLE to allow confidence in changing medications due to its complexity; and (d) feelings of moral obligation or responsibility to others such as family and health care workers for investing time in their care. Themes for not taking SLE medications included: (a) attempting to test whether they were able to control the disease without drugs, due to the belief that SLE could and should be controlled with alternative methods; (b) the belief that long-term use of drugs was unnecessary especially in patients who were well and stable; (c) fear of adverse effects of medications; (d) practical difficulties in obtaining medications such as pharmacy delay in filling the prescription; and (e) poor communication with doctors, for example the physician’s way of introducing new medications to patients, their sensitivity to patients’ questions and concerns, clarity of instructions and frequency of follow-up [31].

Risk factors for medication non-adherence in patients with SLE were investigated by Koneru et al. [32] through patient interviews and medical records review. Results indicated modest adherence to medications: 61% to prednisone, 49% to hydroxychloroquine, and 57% to immunosuppressants. Risk factors for non-adherence included single status (hydroxychloroquine: *χ*^2^ = 4.48; *p* = 0.03; OR = 9.10; 95% CI 1.10–70.3; *R*^2^ = 36%), low educational level (hydroxychloroquine: *χ*^2^ = 4.21; *p* = 0.04; OR = 5.20; 95% CI 1.14–24.0), complicated medication regimens, limited comprehension of physician explanations and instructions (prednisone: *χ*^2^ = 10.70; *p* = 0.001; OR = 26.20; 95% CI 7.30–95; *R*^2^ = 48%; hydroxychloroquine: *χ*^2^ = 6.90; *p* = 0.008; OR = 17.30; 95% CI 4.30–69; *R*^2^ = 53%) and having to take medications more than once daily. A busy lifestyle, forgetting to take the medications or running out of them, and not being at home were the main barriers to adherence reported. In contrast, patients cited pill boxes, tasks lists and better explanation of the rationale of the prescribed medication regimens were most helpful for adhering to medication [32].

## Discussion

The goal for this systematic review was to appraise the evidence identified from published articles relating to physician–patient communication in rheumatology and associated outcomes. The number of papers included in the review was small due to relatively limited published research with a focus on this particular topic. The data suggested that factors associated with doctor–patient communication are distinguished into two categories: (a) more active and positive patient participation in a medical consultation is associated with improved outcomes such as less organ damage, lower disease activity, feelings of being understood, fewer medication side effects, perceptions that the illness is well-controlled, increased decision-making, physician partnership building and supportive talk, greater treatment satisfaction and more positive beliefs about current and future health and (b) higher levels of trust in the physician are linked to better global health, fewer treatment side effects and lower disease activity and increased disclosure of information.

Current results provide some evidence that the quality of communication is linked to improved health outcomes possibly through increased patient participation and trust. For example, one of the most vital health outcomes included organ damage the risk of which is increased with medication non-adherence. As Ward et al. [25] reported, patients who participated more actively in their interaction with physicians tended to have less permanent organ damage which was inversely related in a dose-dependent fashion to more active participation [25]. Patients who were more actively involved in the consultation might have been more likely to obtain information that could have decreased the likelihood of their non-adherence to prescribed medication. For example, greater participation in their care might provide patients with a deeper understanding of ways of the necessity, and side effects of their medication as well as ways to control their condition beyond pharmaceutical agents as Chambers et al. [31] highlighted. In addition, patients who did not have much trust in their physician were more likely to report poorer health [26], while physicians expressing sensitivity to patient concerns and focusing on patient-centredness tended to be linked to fewer medication side effect and better global health [27]. All of these findings are consistent with previous research in the area which showed that rheumatology patients’ HRQoL can be influenced by the nature of their interaction with their physician [2] including the amount of information provided and its association with medication adherence [3].

Identifying the pathways through which communication influences health and well-being particularly in patients with rheumatic diseases is vital to understand why it may lead to better or worse health outcomes. In general, there are two pathways of influence: (a) the direct route, for example, when physician’s behaviour validates the patient’s perspective and expresses empathy for them. This may help a patient experience improved psychological well-being, i.e., less fear or anxiety [42] and (b) the indirect or mediated route, where health status or intermediate outcomes such as adherence, self-management skills or social support could be influenced by mediating factors including satisfaction with care, motivation to adhere to treatment, trust in the clinician, clinician–patient agreement, and shared understanding [1]. Support and clear explanations of disease-related information such as prognosis, treatment options and side effects could improve a particular health outcome, for instance, disease activity through increasing patient trust and understanding which, in turn, might facilitate adherence with the prescribed therapy. Naturally, other factors would need to be considered as well when assessing pathways of influence in communication such as educational level or race [43].

A review by Haskard Zolnierek and DiMatteo [6] showed that training physicians across a variety of specialities in communication skills resulted in substantial and significant improvement in patient adherence with the odds of patient adherence being 1.62 times higher when a physician is trained compared to receiving no training [6]. One of the techniques frequently used to support patients in behaviour change is Motivational Interviewing (MI). MI is a patient-centred counselling technique [44] that can be applied to address key components of long-term disease management such as coping strategies, self-management, medication adherence and can be delivered as part of routine care by a patient’s health care professional [45, 46]. A meta-analysis by Rubak et al. [47] showed that MI was superior to traditional advice-giving in the treatment of a broad range of behavioural problems and diseases. Moreover, both psychologists and physicians obtained an effect in approximately 80% of the studies, while other healthcare providers obtained an effect in 46% of the studies [47].

The primary limitation of this review is the inability to combine the results of studies with varied designs (qualitative, quantitative and mixed methods), interventions, surveys, observational studies, and outcome measures. Particularly, the field of communication research, due to its complex nature, is characterised by a lack of consensus on what to measure [48]. The cross-sectional design of most included studies also prevented establishing causal effects between variables. Moreover, the number of the papers included was not sufficient to allow for strong conclusions to be drawn. This review might also be subject to a slightly higher risk of bias as it had not been pre-registered on PROSPERO. Finally, the majority of quantitative studies were conducted in the US where the health care system is different to models followed in other countries which suggests findings may not be generalisable to other populations.

The lack of more robust evidence and a greater number of studies prevents us from reaching strong conclusions. However, based on the included articles, it can be speculated that patient participation, in concert with greater informational and emotional support provided by the rheumatologist might increase patients’ involvement in their care as well as trust in their doctor which could result in improved outcomes. This would be beneficial for patients, because they might experience better quality of life due to decreased disease activity and organ damage but it may also be cost-effective for the NHS in the long-term due to decreased need for frequent appointments. However, more research is needed to increase our understanding of the dynamics of doctor–patient communication and its impact on outcomes. Thus, future studies could focus on targeting the key elements described in this review and exploring their pathways of influence. Their findings could then be used to inform the design and implementation of appropriate interventions, for instance, physician training in patient-centred counselling techniques such as Motivational Interviewing [44].

To conclude, the current systematic review provides some evidence in support of the importance of the quality of communication between rheumatologists and patients and their relationship to outcomes such as quality of life, medication adherence, and disease activity. Overall, better interaction between the two parties was linked to improved outcomes such as lower disease activity and organ damage, treatment satisfaction and fewer side effects. The two key elements identified in the included studies as maintaining a central role in this were patient participation in the medical consultation and trust in the physician.
